# Characterization and Expression Analysis of the Ca^2+^/Cation Antiporter Gene Family in Tomatoes

**DOI:** 10.3390/plants9010025

**Published:** 2019-12-23

**Authors:** Kayoko Amagaya, Tomoki Shibuya, Manabu Nishiyama, Kazuhisa Kato, Yoshinori Kanayama

**Affiliations:** 1 Graduate School of Agricultural Science, Tohoku University, Aoba-ku, Sendai 980-8572, Japan; 2 Faculty of Life and Environmental Science, Shimane University, Matsue 690-8504, Japan

**Keywords:** *Solanum lycopersicum*, Solanaceae, CaCA superfamily, Ca^2+^/Cation antiporter

## Abstract

The Ca^2+^/cation antiporter (CaCA) superfamily plays an important role in the regulation of the essential element Ca^2+^ and cation concentrations. Characterization and expression analyses of CaCA superfamily genes were performed in the tomato (*Solanum lycopersicum*) as a representative of dicotyledonous plants and fruit crops. Sixteen *CaCA* candidate genes were found and identified as tomato CaCA, *SlCaCA*, by a domain search. In a phylogenetic analysis of the *SlCaCA* superfamily, the 16 genes were classified into *SlCAX*, *SlNCL*, *SlCCX*, and *SlMHX* families. Among them, *Solyc12g011070*, belonging to the *SlCAX* family, had four splice variants, three of which were predicted to be nonfunctional because of a lack of important motifs. EF-hand domains were only found in SlNCL, in addition to consensus Na_Ca_ex domains, and the region containing EF-hand domains was characteristically long in some members of SlNCL. Furthermore, four genes of the *SlCCX* family were found to be intronless. As for intracellular localization, one SlCCX member was predicted to be localized to the plasma membrane, while other SlCCXs, SlCAXs, and SlMHXs were predicted to be localized to the vacuolar membrane. The expression patterns of *SlCaCAs* in various organs, including during several developmental stages of fruit, were classified into four groups. Genes involved in each of the SlCAX, SlNCL, and SlCCX gene families were categorized into three or four groups according to expression patterns, suggesting role sharing within each family. The main member in each subfamily and the members with characteristic fruit expression patterns included genes whose expression was regulated by sugar or auxin and that were highly expressed in a line having metabolite-rich fruit.

## 1. Introduction

Ca^2+^ is an essential element in plants and is important for growth and development, functioning as a second messenger in response to extracellular signaling molecules. The Ca^2+^/cation antiporter (CaCA) superfamily plays an important role in the regulation of Ca^2+^ and cation concentrations in plant cells [1,2,3,4,5,6]. Recently, Singh et al. [7] performed a phylogenetic analysis of CaCA proteins in rice and *Arabidopsis* and proposed that all CaCA proteins should be classified as Na^+^/Ca^2+^ exchangers (NCXs). However, Pittman and Hirschi [8] showed, by phylogenetic analysis and structural modeling, that CaCA contains distinctly different groups with different phylogenies, structures, and functional characteristics. Therefore, not all CaCA proteins can be classified as NCXs. At present, CaCA superfamily proteins in plants are classified into four families: H^+^/cation exchangers (CAXs), Na^+^/Ca^2+^ exchanger-like proteins (NCLs), cation/Ca^2+^ exchangers (CCXs), and Mg^2+^/H^+^ exchangers (MHXs). Taneja et al. [9] showed that CaCA superfamily proteins are classified into the above four families in bread wheat.

Of these four CaCA families, CAX and NCL may play roles in responses to abiotic stress [6,10], and plant hormone signaling and flowering [11,12]. Hocking et al. [13] reported that CAXs may form heteromeric transporters and affect functions of guard cells and mesophyll cells in relation to environmental plasticity. Furthermore, CaCA proteins are considered useful for the production of biofortified crops and phytoremediation because they are involved in metal ion absorption [2,14,15,16]. In addition, several recent reports have focused on functional analyses of CAX1 in the CaCA family. Navarro-Leon et al. [17] and Qiao et al. [18] reported that CAX1 is involved in the transport of Ca^2+^ and tolerance for heavy metals in *Brassica* plants and diploid wheat relatives by using mutants and overexpressors. Ahmadi et al. [19] showed that CAX1 suppresses the formation of Cd-induced reactive oxygen species as a stress tolerance mechanism. These various reports indicate the multifunctionality of the CaCA family genes in plants.

Tomatoes (*Solanum lycopersicum*) are an important crop worldwide because of their high economic and nutritional value. They are also scientifically useful as an experimental model species of the Solanaceae family and of fleshy-fruited plants. Regarding tomato CaCA members, it has been reported that the tomato CAX, LeCAX2, transports Ca^2+^ and Mn^2+^ [20], and its expression is increased by gibberellin (GA_4+7_) [21]. Ca^2+^ is closely related to the occurrence of a serious physiological disorder called blossom-end rot in tomato plants, and the relationship between this physiological disorder and CaCA may be interesting [22,23]. However, to our knowledge, tomato NCL and CCX gene families have not yet been reported, and information on tomato CaCA is lacking. In addition, the CaCA superfamily has only been comprehensively characterized in *Arabidopsis* and cereals such as rice and bread wheat [7,8,9], while information on dicotyledonous plants and fruit crops is lacking. Therefore, in order to obtain basic knowledge of CaCA, comprehensive characterization of the CaCA superfamily was performed with expression analysis in tomato plants. Together with previous reports, the present study shows the general and unique properties of the CaCA gene family. In addition, for the first time, by using bioinformatics, we use the results of this study to show analysis examples of the main member in each CaCA subfamily and the members with characteristic fruit expression patterns.

## 2. Results and Discussion

### 2.1. Phylogenetic Analysis of the Tomato CaCA (SlCaCA) Gene Family

Based on a BLAST search using the CaCA sequences of *Arabidopsis thaliana*, it was estimated that 16 CaCA genes exist in the tomato plant. Phylogenetic analysis showed that the 16 putative CaCA proteins could be classified into four families: six into the CAX family (*Solyc03g123790*, *Solyc06g006110*, *Solyc07g056110*, *Solyc09g005260*, *Solyc12g011070*, and *Solyc12g055750*), four into the NCL family (*Solyc02g077270*, *Solyc03g006260*, *Solyc07g062700*, and *Solyc12g014110*), five into the CCX family (*Solyc01g098800*, *Solyc02g069710*, *Solyc07g006370*, *Solyc07g042000*, and *Solyc09g072690*), and one into the MHX family (*Solyc06g009130*). The Sol Genomics Network has published the nucleotide sequence and amino acid sequence of cv. Heinz 1706, although Saand et al. [24] reported sequence errors in the Sol Genomics Network database and the sequence is considered to differ between cultivars [25]. Therefore, the nucleotide sequence of the open reading frame of each CaCA gene was determined in the present study based on cDNA from cv. M82 with primers designed using the nucleotide sequence of each putative CaCA.

**Figure 1:**
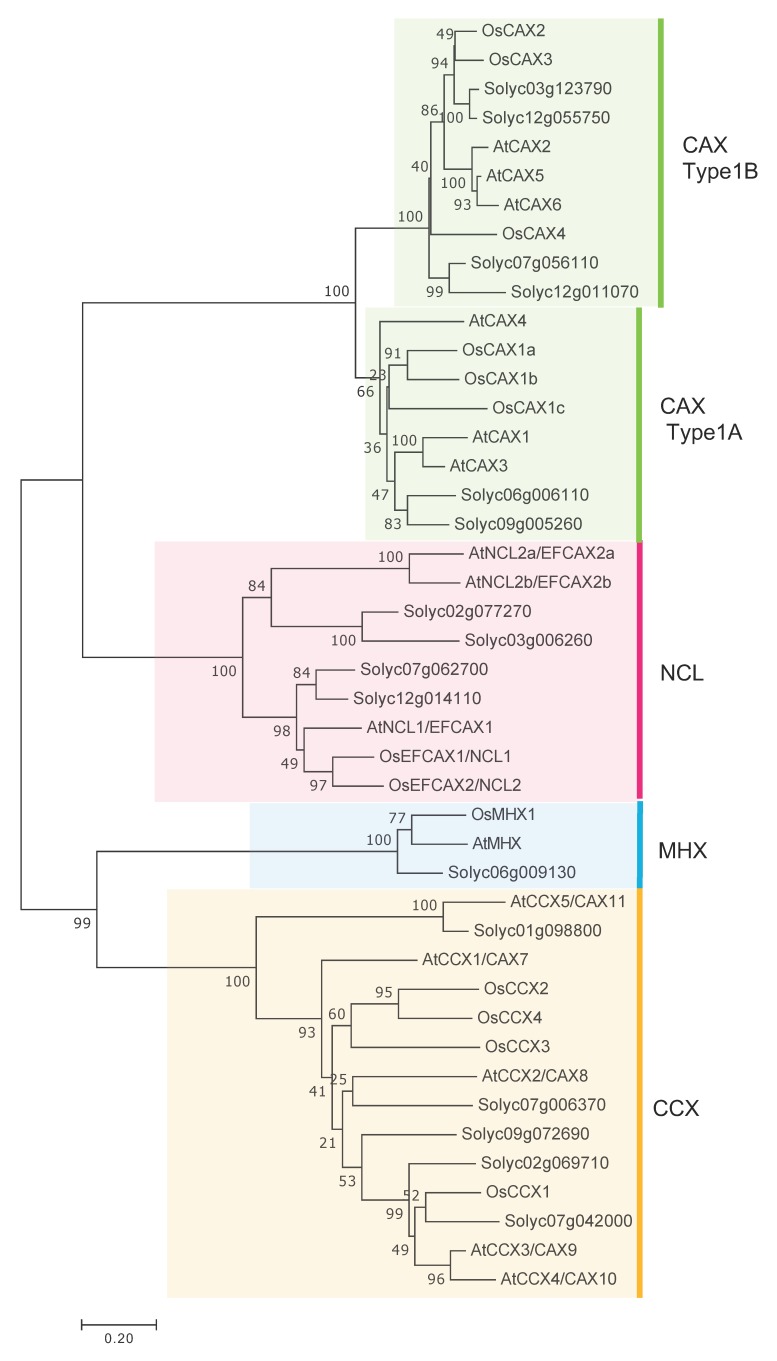
Phylogenetic tree based on amino acid sequences in the Ca^2+^/cation antiporter (CaCA) superfamily of the tomato, *Arabidopsis*, and rice. The phylogenetic tree was built using the neighbor-joining method by MEGA7. Branch numbers are the percentages of replicates that support the branch using the bootstrap method (1000 replicates). The scale bar corresponds to 0.2 amino acid substitutions per residue. The sequences from *Arabidopsis* and rice were obtained from Emery et al. [26] and Pittman and Hirschi [8].

Phylogenetic tree analysis was performed using the amino acid sequences of CaCA genes from the tomato, which were determined here, with those previously determined from rice and *Arabidopsis* [8,26], as shown in Figure 1. As a result, 16 CaCAs were grouped into four families: six CAXs, four NCLs, five CCXs, and one MHX, similar to the results of putative tomato CaCAs. The CAXs were further classified into Type1A and Type1B. According to Emery et al. [26] and Shigaki et al. [27], plant CAXs can be classified as Type1A and plant and moss CAXs can be classified as Type2B, suggesting that there are functional differences between Type1A and Type1B. According to Hirschi et al. [2], Shigaki et al. [28], and Edmond et al. [20], Type1B AtCAX2 and AtCAX5 transport various cations, including Ca^2+^, Cd^2+^, and Mn^2+^ [2,20,28], and Type1A AtCAX1 and AtCAX3 specifically transport Ca^2+^ in *Arabidopsis* [28,29]. However, there are other reports that Type1A CAXs have a broad cation specificity [3,27,30].

According to Emery et al. [26], the average number of CaCA genes per species is 5.8 in algae, while it is 13.25 in land plants. Therefore, the tomato, with its 16 genes, has an above average number of CaCA genes for land plants. As shown in Figure 1, the number of genes in each subfamily was similar in the three compared species. On the other hand, according to Emery et al. [26], NCL (EFCAX) shows the most diversity in the number of genes, with 1 to 5 genes in land plants. In the species used in this study, the number of NCL genes was 2 to 4 and was largest in the tomato, one of which was *Solyc07g062700*. As discussed later, this gene is of interest because of its expression pattern in fruit, the fact that it exhibits the highest expression among SlNCL genes, its response to sugar signals, and its high expression in fruit rich in metabolites. Functional analysis is necessary for both plant physiology and agricultural science.

### 2.2. SlCaCA Protein and Gene Characterization

In order to confirm whether the putative *SlCaCAs* of the tomato function as CaCAs, a domain search was performed on the obtained amino acid sequence using TMHMM (ver. 2.0) and InterPro of the EMBL-EBI. The four mRNA variants of *Solyc12g011070* were referred to as *Solyc12g011070a*, *b*, *c*, and *d*. CaCA has transmembrane domains (TMs) because it performs the countertransport of cations through the membrane. According to previous reports, CaCA has an average of 10 TMs [8,9,26]. However, in the amino acid sequence translated from *Solyc12g011070b*, *c*, and *d*, there were only five TMs (Figure S1). Excluding these variants, the number of TMs in the estimated CaCA was 9 to 13, and the average was 10.9, which is, in general, in agreement with the previous report (Figures S1–S3; Table 1).

**Figure 2:**
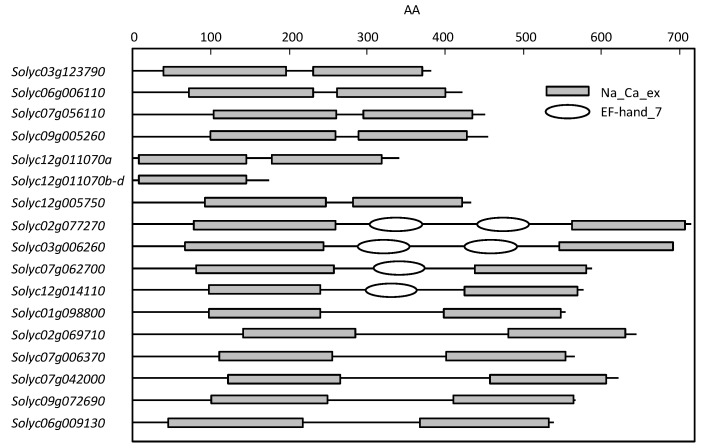
Domain architecture of tomato CaCA (*SlCaCA*) proteins. Gray rectangles and white ovals represent Na_Ca_ex and EF-hand domains, respectively.

When the domain was searched with InterPro of the EMBL-EBI, the amino acid sequences translated from *Solyc12g011070b*, *c*, and *d* had structures different from those documented in previous reports and other putative tomato CaCAs (Figure 2). That is, only one Na_Ca_ex domain (Pfam ID, Pf01699; [9]), which is characteristic of CaCA, was present in *Solyc12g011070b*, *c*, and *d*, while two were present in other CAXs, NCLs, CCXs, and the MHX. In addition, only NCLs contained EF-hand domains (Pfam ID: PF13499): two in *Solyc02g077270* and *Solyc03g006260* and one in *Solyc07g062700* and *Solyc12g014110*. According to Taneja et al. [9], CaCAs in bread wheat have two Na_Ca_ex (Pfam ID: Pf01699) domains, and only TaNCL has one EF-hand domain (Pfam ID: PF13499). As described above, *Solyc12g011070b*, *c*, and *d* did not have domains of importance to CaCA, so they were not considered to be functional, unlike CaCA, and were excluded from expression analysis. However, since expression regulation by selective splicing has been reported in the stress response [31], it may be necessary to examine the role of variant generations of this gene. Despite this, since important domains were conserved in other putative CaCAs, it was concluded that there were 16 functional CaCAs in the tomato, and these were categorized as SlCAX, SlNCL, SlCCX, or SlMHX.

Emery et al. [26] examined a wide range of organisms, including plants, and found that, although not classified as NCL, some CaCA members in mainly land plants have an additional EF-hand domain with a Ca-binding motif to Na_Ca_ex domains, which is a consensus sequence. Taneja et al. [9] showed that in TaNCL of wheat TaCaCA, the EF-hand domain exists as a loop that is a long amino acid sequence between two TMs near the center, and it is longer than those of members in other subfamilies. This also matched the tomato CaCA superfamily; additional EF-hand domains, specific to SlNCL, were found in long amino acid sequences between the specific TMs (Figure 2 and Figures S1–S3). However, members with one or two EF-hand domains were found in the tomato, and among them, members with two were particularly characteristic because the amino acid sequence between the corresponding TMs was nearly twice the length of that of TaNCL. Therefore, future studies should demonstrate the significance of this in function and evolution.

**Figure 3:**
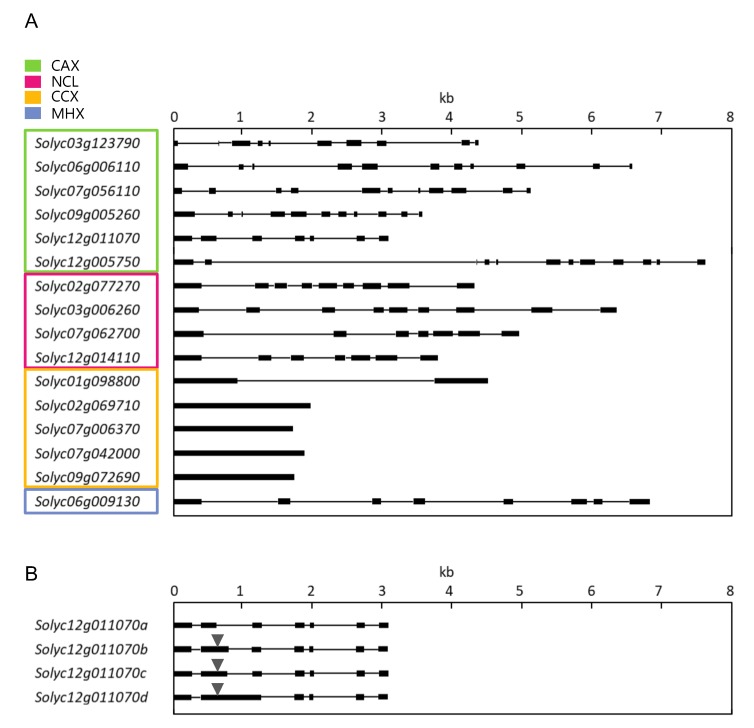
Intron and exon structure of *SlCaCA* superfamily genes. Exons and introns are shown as black boxes and black lines, respectively. The structure of 16 *SlCaCA* genes includes that of functional variant a of *Solyc12g011070* (**A**). The structures corresponding to four mRNA variants of *Solyc12g011070* are also shown as variants a–d, with arrowheads indicating premature stop codons (**B**).

To examine the genomic structure of each CaCA, schematic diagrams of exons and introns were made, based on the genomic DNA sequences of the corresponding genes (European Nucleotide Archive, https://www.ebi.ac.uk/ena) (Figure 3). The second exons of *Solyc12g011070b*, *c*, and *d* contained part or all of the second intron of *Solyc12g011070a*. Because a stop codon appears in the second intron of *Solyc12g01107a* (523 bases from the start codon), the translated regions of *Solyc12g011070b*, *c*, and *d* were half the length of the functional sequence of *Solyc12g011070a*, and consequently contained only one Na_Ca_ex (Pfam ID: Pf01699) domain. *Solyc09g005260* and *Solyc02g069710* were different in sequence from cv. Heinz 1706. However, it was likely that there was no difference in their functions as CAXs because the difference in the sequence was not within the important motif. *Solyc02g069710*, *Solyc07g006370*, *Solyc07g042000*, and *Solyc09g072690*, belonging to CCX, had no introns. Three of five TaCCXs also lack introns in bread wheat [9], suggesting that this may be a feature of CCX that is conserved across species and across dicotyledonous and monocotyledonous plants. Because intronless genes form mature mRNAs without splicing, they can respond quickly to stress [32,33]. This suggests that CCX is involved in the stress response, and that transcription and translation of these genes may occur rapidly due to the absence of introns. On the other hand, since there are many intronless genes involved in basic cellular processes [33,34], the intronless genes may have significance, such as avoiding energy loss during the transcription process, other than the quick stress response.

### 2.3. Protein Structure and Localization

The amino acid sequences of the tomato α1-repeat and α2-repeat regions were compared with those of rice and *Arabidopsis*, with reference to Kamiya et al. [3] and Taneja et al. [9]. The regions called α1-repeat and α2-repeat conserved in rice and *Arabidopsis* are motifs characteristic of CaCA, and they are considered to be cation-binding regions [35,36,37,38]. The α1-repeat and α2-repeat sequences of SlCAX, SlCCX, and SlMHX were very common among species (Figures S4–S6). According to previous reports, however, NCL seems to contain the α2-repeat, but not the α1-repeat [8,9,25]. In the present study, the sequences of the region possibly corresponding to the α1-repeat of SlNCL were compared among species, and common sequences were found, in particular, in *OsEFCAX1/NCL1*, *OsEFCAX2/NCL2*, *Solyc07g062700*, *Solyc12g01411*, and *AtNCL1/EFCAX1*, which clustered together in the phylogenetic tree (Figure 1 and Figure S7). The function of this region remains to be established.

An α1-repeat was found in the region containing TM1-2, TM2-3, or TM3-4, and an α2-repeat was found in the region containing TM6-7, TM7-8, TM8-9, or TM9-10 (Table 1). When the number of TMs was 10, an α1-repeat was found in the region of TM2-3 and an α2-repeat was found in the region of TM7-8, which is in agreement with previous reports [8,9,25]. According to TMHMM, *Solyc01g098800* (SlCCX) and *Solyc02g069710* (SlCCX) contain an α1-repeat in the region containing only TM3, whereas, according to Taneja et al. [9], an α1-repeat is present in the region containing TM2-3. According to TMHMM, *Solyc06g009130* (SlMHX) contains an α2-repeat in the region containing TM9-10, whereas, according to Taneja et al. [9], an α2-repeat is present in the region containing TM7-8. In other CaCAs with two α-repeats, these had TM helices facing each side of the membrane. In summary, the α-repeat regions of *Solyc01g098800*, *Solyc02g069710*, and *Solyc06g009130* were determined with reference to Taneja et al. [9].

Identifying intracellular localization is important to understanding how CaCA regulates the cation concentration in cells. Therefore, the intracellular localization of CaCAs was predicted by ProtComp (Table 1). It was predicted that all SlCAXs localized to the vacuolar membrane, while the localization of SlNCL could not be predicted. Among SlCCXs, *Solyc01g098800* was predicted to be localized to the plasma membrane, while other S1CCXs were predicted to be localized to the vacuolar membrane. It has been reported that CaCAs localize to vacuolar and plasma membranes [7,39]; the localization prediction here is consistent with those reported previously. In addition, AtCAX1, AtCAX2, AtMHX, and OsCCX2 have been experimentally confirmed to be localized to the vacuolar membrane [2,5,40,41], and tomato CaCAs contained in the same cluster as AtCAX1 and AtMHX in the phylogenetic tree were also predicted to be localized to the vacuolar membrane in the present study. On the other hand, AtCCX5, which is classified as being in the same CCX group as *Solyc01g098800*, has been experimentally confirmed to be localized to the plasma membrane [42].

### 2.4. Expression Profile of SlCaCA Genes in Vegetative Organs, Flowers, and Fruit throughout Development

**Figure 4:**
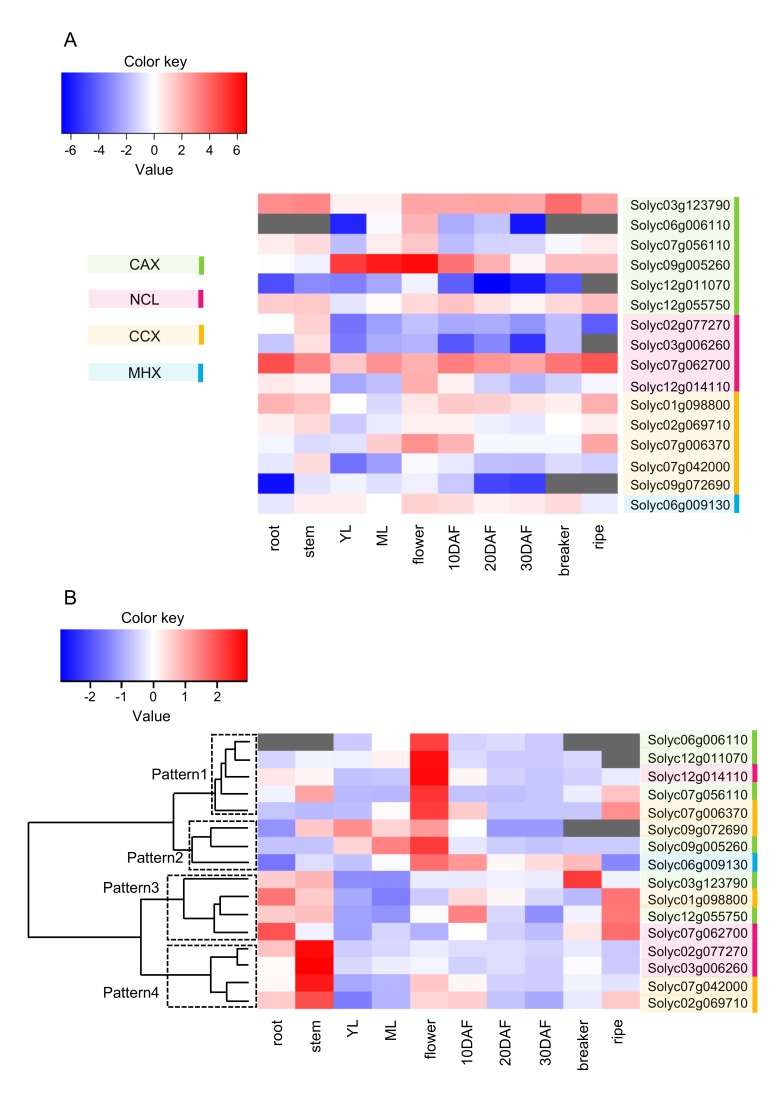
Expression profile of *SlCaCA* superfamily genes in various organs containing fruit from 10 days after flowering (DAF) to ripeness. YL and ML represent young and mature leaves, respectively. (**A**) In order to accurately compare the expression levels between genes, the copy number corresponding to the amount of each mRNA was determined using the standard curve. The expression level was expressed as log_2_, and the heat map was prepared by subtracting the expression level of each gene from the median; red represents the highest expression levels, white indicates median expression, and blue shows the lowest expression levels. Gray represents undetectable expression. Each log_2_ expression value was based on the mean of three biological replicates. (**B**) To compare the expression levels among organs for each gene, expression levels were normalized in each gene. Color bar denotes Z-score: red represents the highest expression levels, white indicates mean expression, and blue shows the lowest expression levels in each gene. Gray represents undetectable expression. Hierarchical clustering was performed using Ward’s method. Each expression value was based on the mean of three biological replicates.

Although the expression profile can be referred to in the web database, the expression differs depending on the variety, cultivation condition, and measuring method. In fact, the expression pattern was similar, but sometimes different, from that of the web database. Therefore, expression analysis was carried out during this study. A heat map of the expression levels of 16 genes in various organs and some developmental stages of fruit is shown in Figure 4A, in which expression levels can be appropriately compared between genes. There were two genes (*Solyc12g011070* and *Solyc09g072690*) for which expression was not detected at all or was lower than the median if detected. However, the mRNA levels of other genes were above the median in one or more of the organs and fruit developmental stages. Therefore, in order to clarify the characteristic expression patterns for each gene, expression levels in organs and fruit developmental stages were normalized, and hierarchical clustering was performed based on the expression patterns (Figure 4B). As a result, expression patterns were divided into two clusters: high expression in flowers and high expression in fruit, stems, or roots with low expression in leaves. The former was further divided into two clusters: high expression specifically in flowers (Pattern1) and relatively high expression in other organs, as well as flowers (Pattern2). The latter was also divided into two clusters: high expression in fruit, roots, and stems (Pattern3) and low expression in fruit, with the highest expression in stems (Pattern4). There were only two genes with a higher than average expression in leaves, suggesting that CaCAs are more important in sink organs in constitutive expression. Since *SlCAXs*, *SlNCLs*, and *SlCCXs* were classified into three, three, and four different patterns, respectively, each gene belonging to the same family may play different roles in various organs and developmental stages of fruit.

For Pattern3 genes, in which the expression is high in fruit, expression was commonly high 10 days after flowering (DAF) and during breaker and ripe stages. The stages 10 DAF, 20 to 30 DAF, and breaker to ripe correspond to cell division, cell expansion, and ripening stages, respectively [43], and Pattern3 genes may play a role in the cell division and ripening stages. *TaCAX* from bread wheat shows a high expression in grains and seeds [9], and tomato *CAX*, *SlCAX*, in Pattern3, *Solyc03g123790*, and *Solyc12g055750*, also showed a high expression during the fruit ripening stage, suggesting a similar role for those genes. CAX is mainly present in the vacuolar membrane and is important in H^+^/Ca^2+^ exchange, according to previous reports [44,45]. As for the relationship between fruit ripening and Ca^2+^, Ca^2+^-mediated cross-linking of pectin accompanied by demethylation by pectin methylesterase has been recorded [46], and the transport of Ca^2+^ into vacuoles by SlCAX might affect this ripening-related phenomenon. In fact, it has been reported that overexpression of the *Arabidopsis* CAX gene in the tomato reduces the Ca^2+^ concentration in the fruit apoplastic fraction, which contains cell wall pectin [47].

Pattern3 also included *Solyc01g098800*, belonging to *SlCCX*. CCX localizes to the vacuolar or plasma membrane [4,42,48], and, in *Arabidopsis*, AtCCX1 and AtCCX3 play roles in Na^+^/K^+^ exchange and H^+^/K^+^ and Na^+^ exchange, respectively [4,48]. AtCCX5 plays a role in K^+^ uptake [42]. *Solyc01g098800* was closest to AtCCX5 in the phylogenetic tree (Figure 1) and was predicted to be localized to the plasma membrane like AtCCX5, suggesting its role in K^+^ uptake. Tomato fruits are rich in K^+^. While K^+^ intake is useful for preventing hypertension [49], it is necessary to suppress intake in renal dysfunction [50]. Therefore, control of the K^+^ concentration related to SlCCX is an important issue.

**Figure 5:**
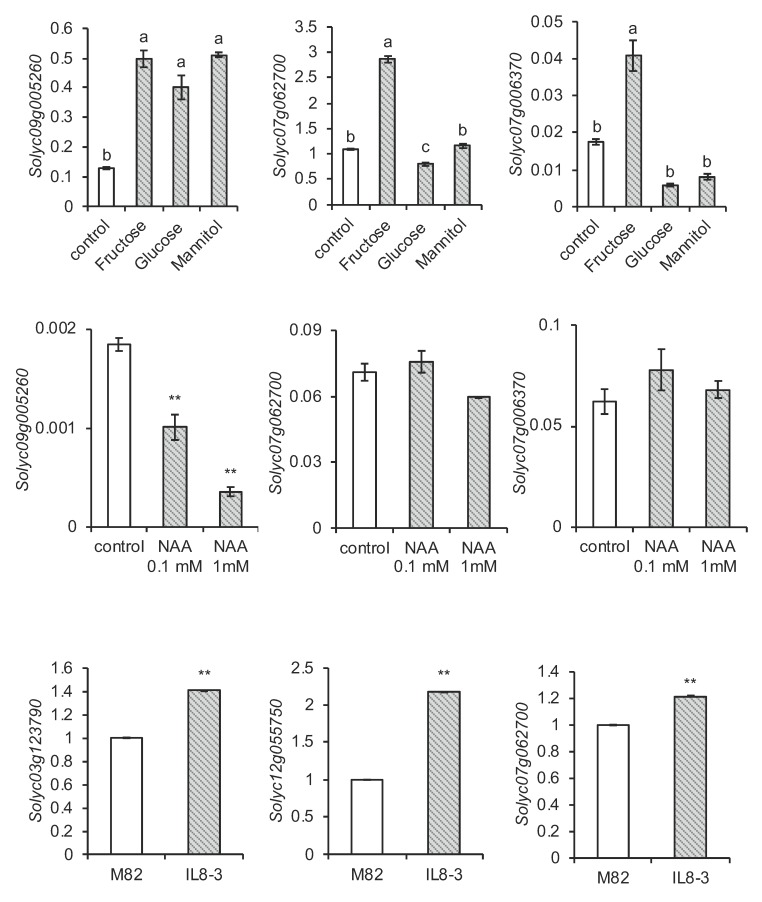
In the top two rows, the effect of sugar and auxin (NAA) on gene expression is shown for *Solyc09g005260 (SlCAX)*, *Solyc07g062700 (SlNCL)*, and *Solyc07g006370 (SlCCX)*. Relative expression levels are shown, with error bars indicating the standard error of the mean of three biological replicates. Values with different letters are significantly different at *P* < 0.05 according to the Tukey–Kramer test. In the bottom rows, the expression of *Solyc03g123790 (SlCAX)*, *Solyc12g055750 (SlCAX)*, and *Solyc07g062700 (SlNCL)* is shown for fruits of parental tomato cultivar M82 and its near-isogenic line IL8-3 containing a chromosome segment from tomato’s wild relative. Relative expression levels from the microarray data [51] are shown, with error bars indicating the standard error of the mean of three biological replicates. ** *P* < 0.01 between M82 and IL8-3, as revealed by a *t*-test.

We showed the main members in each CaCA subfamily and members with characteristic fruit expression patterns for the first time by genome-wide studies of the CaCA family in fruit crops; thus, various analyses using this information became possible. We focused on the expression of these genes in response to sugar and auxin, as well as in fruit rich in metabolites, since there is a lack of information on the regulation of expression other than the stress response. Because the highly expressed genes in the three subfamilies were *Solyc09g005260* in *SlCAX*, *Solyc07g062700* in *SlNCL*, and *Solyc07g006370* in *SlCCX* (Figure 4A), these genes were hypothesized to play a central role in each family. Sugar is known to affect the metabolism as an important signal in fruit [51,52]. Therefore, the induction of expression by sugar was examined for these three genes. An enhanced expression of *Solyc09g005260 (SlCAX*) was induced by fructose, glucose, and mannitol (Figure 5). In contrast, although *Solyc07g062700* (*SlNCL)* and *Solyc07g006370 (SlCCX)* expression was remarkably upregulated by fructose treatment, it was slightly downregulated or unchanged by glucose and mannitol. If gene expression is affected by mannitol, as well as fructose and glucose, as with *Solyc09g005260*, it is thought to be a response to osmotic stress; however, the expression of *Solyc07g062700* and *Solyc07g006370* appeared to occur in response to the sugar signal instead of osmotic stress. Interestingly, *Solyc07g062700*, an *SlNCL*, belonged to Pattern3 (Figure 4B), which suggests its importance in fruit. Its expression in ripe fruit was the highest of all genes, as shown in Figure 4A. Its expression level increased during fruit development, a pattern consistent with that of sugar accumulation in the tomato fruit [53]. Because fructose and glucose rely on different sensors of hexokinase 1 (HXK1) and fructose-insensitive 1 (FINS1), respectively [54], the response of the gene may be different for fructose and glucose. In fact, glucose is more effective in the enhancement of glutamate synthase gene expression, in contrast to *Solyc07g062700* [51]. The role of *Solyc07g062700* in fruit metabolism requires further investigation.

Regarding the relationship between auxin and CaCA, Li et al. [12] reported that auxin suppresses NCL expression in *Arabidopsis*. In this study, *SlNCL* (*Solyc07g062700)* expression was not affected by auxin (1-naphthylacetic acid, NAA), whereas *SlCAX (Solyc09g005260*) expression was suppressed (Figure 5). Using *Arabidopsis* mutants, Cho et al. [11] reported that CAX promotes apoplast pH reduction. Since apoplast pH reduction may affect auxin transport and cell elongation, which are related to fruit development, the regulation of *SlCAX* expression by auxin in this study could be interesting. In fact, auxin affects cell division, cell size, and the expression of genes related to them in tomato fruit [55].

IL8-3 is a near-isogenic line in which a chromosomal fragment of tomato’s wild relative is introduced into chromosome 8 [56]. The fruit of IL8-3 shows a useful phenotype with high sugar and amino acid concentrations, which is derived from metabolism in the fruit at 20 DAF [51]. Therefore, we compared the expression of the Pattern3 genes, which were predicted herein to play a role in the fruit, between IL8-3 and the parental cultivar M82 at 20 DAF using the published omics data [51]. The mRNA levels of the three genes analyzed were higher in IL8-3 (Figure 5), suggesting a relationship between the CaCA genes and the active metabolism of IL8-3 fruit.

MHX is thought to be highly expressed in stems and leaves as it transports Mg^2+^, which is important for chlorophyll synthesis [9]. In the organs analyzed in the present study, the expression of *SlMHX* was indeed lowest in roots and ripe fruit, which did not contain chlorophyll (Figure 4B), and lower than the median (Figure 4A). All other organs, including flowers with green sepals, contained chlorophyll, supporting the previous report. However, since AtMHX in *Arabidopsis* transports Zn^2+^ and Fe^2+^, as well as Mg^2+^ [5], it is necessary to consider whether it has roles other than in chlorophyll synthesis.

## 3. Materials and Methods

### 3.1. Plant Materials

*Solanum lycopersicum* cv. M82 plants were grown in a greenhouse, as described previously [51], and their roots, stems, young leaves, mature leaves, flowers, and fruit were used for analysis of the sequence of *SlCaCA* cDNA and its expression (Figure 4). Fruit was sampled at 10 DAF, 20 DAF, 30 DAF, the beaker stage, and the ripe stage, respectively. *Solanum lycopersicum* cv. Ailsa Craig plants were used to investigate the effects of sugar and auxin on gene expression (Figure 5). For sugar treatment, cotyledons 5 days after germination were incubated with 300 mM glucose, fructose, or mannitol and sampled as described previously [51]. For auxin treatment, tomato plants were grown in a phytotron, and fruit was dipped in 0.1 mM or 1 mM NAA every day from 4 DAF, as described previously, and used at 15 DAF for expression analysis [55].

### 3.2. RNA Extraction and cDNA Synthesis

RNA was extracted using a Cica Geneus RNA Prep Kit (for Plant, Kanto Chemical). ReverTra Ace^®^ qPCR RT Master Mix (TOYOBO) was used for genomic DNA removal and the reverse transcription reaction to prepare cDNA.

### 3.3. SlCaCA Sequence Analysis

A BLAST search was performed in the National Center for Biotechnology Information (http://www.ncbi.nlm.nih.gov/) and the Sol Genomics Network (http://solgenomics.net/) to search for putative *SlCaCA* proteins using *Arabidopsis thaliana* CaCA (AtCaCA) amino acid sequences identified by Emery et al. [26] and Pittman and Hirschi [8]. Using the sequences of *Arabidopsis* CaCAs, homologous sequences having a query cover of approximately 100 and the highest identity were selected. Furthermore, the BLAST search was performed again using the obtained sequence as a query to confirm the presence or absence of similar sequences. The presence or absence of similar sequences was also checked, taking into account the possible presence of tomato CaCA that does not correspond to each member of *Arabidopsis* CaCA. Specific primers were designed for each putative *SlCaCA* gene based on the sequence of *S. lycopersicum* cv. Heinz 1706 to amplify the cDNA by PCR with Q5 High-Fidelity DNA Polymerase (New England Biolabs, Japan). Primer sequences and annealing temperatures are shown in Table S1. The PCR product was electrophoresed on 1% agarose gel and extracted using NucleoSpin^®^ Gel and PCR Clean-up (Mahala Nagel). Extracted DNA and primers were submitted to Macrogen, Japan, for general sequence analysis.

Multiple sequence alignments using amino acid sequences of *SlCaCA* and *A. thaliana* and *Oryza sativa* CaCA identified by Emery et al. [26] and Pittman and Hirschi [8] were made using Clustal W and an unrooted neighbor-joining tree was constructed based on a full-length protein sequence with 1000 bootstrap replications by MEGA7 software [57]. A domain search was performed using TMHMM (ver. 2.0, http://www.cbs.dtu.dk/services/TMHMM/) and EMBL-EBI InterPro (https://www.ebi.nih.gov) on *SlCaCA* amino acid sequences to define CaCA proteins based on important conserved domains. Subcellular localization was examined using ProtComp (ver. 9.0, http://www.softberry.com/berry.phtml? Topic = protcomppl & group = programs & subgroup = proloc). The protein and genomic structures of Figure 2 and Figure 3 were constructed with PowerPoint (Microsoft) based on length after analyzing the sequence with the software described above and Genetyx (ver. 10; https://www.genetyx.co.jp/).

### 3.4. Expression Analysis

For real-time PCR, KOD SYBR qPCR Mix (TOYOBO) was used with primers at the annealing temperatures described in Table S2. PCR was performed using a PTC-2000 DNA Engine Cycler CFX Connect Real-Time Detection System (Bio Rad), according to the following protocol: 98 °C for 2 min; 39 cycles of 98 °C for 15 s, X °C for 30 s, and 68 °C for 30 s, plate read; melting curve from 65 °C to 95 °C, read every 0.2 °C, hold for 10 s. A melting curve was used to confirm the presence of single products.

To accurately compare the expression levels between genes in Figure 4A, the copy number corresponding to the amount of each mRNA was determined using a standard curve obtained by employing the copy number calculated from the cDNA fragment of each *SlCaCA* and *SlUbiquitin* of known concentrations. The cDNA fragments were extracted from the agarose gel mentioned in Section 3.3, and the standard curves were prepared based on the calculated copy numbers and the results of real-time PCR using a series of cDNA with a known copy number in each gene. The amount of mRNA was shown as the ratio of the copy number of the target gene to that of *SlUbiquitin*. To compare the expression levels among organs for each gene in Figure 4B, expression levels were normalized in each gene.

For expression analysis of the sugar and auxin response in Figure 5, calibration using the standard curves was not performed. Threshold cycles were determined, and the constitutively expressed ubiquitin gene was used as a reference for the normalization of gene expression. The levels of DNA microarray signals [51] were used to show the relative abundance of transcripts of Pattern3 genes (*Solyc03g123790*, *Solyc12g055750*, and *Solyc07g062700*) in M82 and its near-isogenic line IL8-3 in Figure 5. The microarray was designed for 43,803 tomato probe sets based on the tomato whole-genome sequences. The data for *Solyc01g098800*, another gene in Pattern3, was not shown in Figure 5 because the gene had been removed in a false discovery rate selection. The data were analyzed with the Tukey–Kramer test or Student’s t test using the Excel Toukei ver. 3.0 (Social Survey Research Information Co., https://www.ssri.com/).

## 4. Conclusions

The structure and expression of CaCA superfamily genes were examined in the tomato, as a representative of dicotyledonous plants and fruit crops. Sixteen CaCA candidate genes were found, identified as CaCA, and classified into four subfamilies, and we identified their general and unique properties. The expression patterns of CaCAs were classified into four groups. The main members in each subfamily and the members with characteristic fruit expression patterns were revealed for further studies to elucidate the roles of CaCA family genes. Similar bioinformatics reports on other tomato transporters have had a significant impact on the many citations [58,59], and by integrating these types of studies and functional genomics, we may reveal the whole picture of fruit metabolism in the future.

## Figures and Tables

**Table 1:** Characteristic features of tomato CaCA superfamily proteins.

Family	Locus ^a^	Protein Size ^b^	TM ^c^	Domain ^d^	α1-Repeat ^c^	α2-Repeat ^c^	Localiza-tion ^e^
CAX	*Solyc03g123790*	383	11	Na_Ca_ex	TM3-4	TM8-9	vacuole
	*Solyc06g006110*	423	11	Na_Ca_ex	TM3-4	TM8-9	vacuole
	*Solyc07g056110*	452	10	Na_Ca_ex	TM2-3	TM7-8	vacuole
	*Solyc09g005260*	456	10	Na_Ca_ex	TM2-3	TM7-8	vacuole
	*Solyc12g011070a*	342	9	Na_Ca_ex	TM1-2	TM6-7	vacuole
	*Solyc12g011070b*	175	5	Na_Ca_ex	TM1-2		
	*Solyc12g011070c*	175	5	Na_Ca_ex	TM1-2		
	*Solyc12g011070d*	175	5	Na_Ca_ex	TM1-2		
	*Solyc12g055750*	434	11	Na_Ca_ex	TM3-4	TM8-9	vacuole
NCL	*Solyc02g077270*	716	10	Na_Ca_ex, EF-hand		TM7-8	
	*Solyc03g006260*	694	10	Na_Ca_ex, EF-hand		TM7-8	
	*Solyc07g062700*	589	11	Na_Ca_ex, EF-hand		TM8-9	
	*Solyc12g014110*	578	10	Na_Ca_ex, EF-hand		TM7-8	
CCX	*Solyc01g098800*	555	11	Na_Ca_ex	TM2-3 ^f^	TM9-10	plasma membrane
	*Solyc02g069710*	646	11	Na_Ca_ex	TM2-3 ^f^	TM9-10	vacuole
	*Solyc07g006370*	567	13	Na_Ca_ex	TM3-4	TM10-11	vacuole
	*Solyc07g042000*	623	13	Na_Ca_ex	TM3-4	TM10-11	vacuole
	*Solyc09g072690*	568	12	Na_Ca_ex	TM2-3	TM9-10	vacuole
MHX	*Solyc06g009130*	540	11	Na_Ca_ex	TM2-3	TM7-8	vacuole

^a^ Sol Genomics Network database. ^b^ Amino acids. ^c^ Number of transmembrane domains and α-repeat regions (TMHMM). ^d^ EMBL-EBI. ^e^ ProtComp. ^f^ According to Taneja et al. [9].

## Supplementary Materials

The following are available online at https://www.mdpi.com/2223-7747/9/1/25/s1: Figure S1: Transmembrane domains of SlCAX searched using TMHMM; Figure S2: Transmembrane domains of SlNCL searched using TMHMM; Figure S3: Transmembrane domains of SlCCX and SlMHX searched using TMHMM; Figure S4: Multiple alignments of conserved α1-repeat and α2-repeat regions in CAX proteins; Figure S5: Multiple alignments of conserved α1-repeat and α2-repeat regions in CCX proteins; Figure S6: Multiple alignments of conserved α1-repeat and α2-repeat regions in MHX proteins; Figure S7. Multiple alignments of conserved and α2-repeat regions in NCL proteins; Table S1: List of primers used for sequence analysis of *SlCaCA*; Table S2: List of primers used for expression analysis of *SlCaCA*.
