# Supplementary material for: Characterization and Expression Analysis of the Ca2+/Cation Antiporter Gene Family in Tomatoes
Source: Plants (Basel). 2019 Dec 23;9(1):25. doi: 10.3390/plants9010025 (PMC7020160; doi:10.3390/plants9010025)
Supplement: Supplementary file 1 [file plants-09-00025-s001.pdf]

Table S1. List of primers used for sequence analysis of *SlCaCA*.

Family name	Locus by ITAG 2.4	Gene symbol by NCBI	Primer sequence(5'→3') Forward	Reverse	Annealing temp.(℃)
CAX	*Solyc03g123790*	LOC101267212	CCATTTTCAGAACAGCCGGC	AGTCGAGGTGCTCATTTGGC	68
	Solyc06g006110	LOC101265339	GGGATTTGGAGCATAATGTTGG	CGGTTGATGACGTGTACCA	65
			CCCCTATTAAATCAACATTTTCTAGTCC	GTTCAGTCAAGAAGCTGACACG	64
			GGTACGATCGAGGCTGCATCAG	GCACCAATGACACAATAGGCAAGAC	69
	Solyc07g056110	LOC101252790	CGTAAGCTACGCCAAAGCAA	CAGCTCAGAGTGGCCAAAAC	67
	Solyc09g005260	LOC101254519	GGCTTCATCATCACCAGCA	GGAACTCTCAGCAACTATGGA	65
			CCCCATACTCATACAAGATGAGA	TTGATGAGCCCAAATTGACA	64
	Solyc12g011070	—	GCCAATATCATCTCGATTAATCTGC	GCGAGGGAGGAAACTTACG	64
			ATGGCACTCTATTTAGCCAATATC	CTACCATATAATAATTGTATGAAGCGAG G	64
	Solyc12g055750	LOC101250779	ACGATTCGATTCGCCCACTG	ACTGCCAGTTGTTGAGGAGTTC	68
NCL	*Solyc02g077270*	LOC101251749	CTTTGGTAGTGATGCCAAGCC	TGAATGGGTTTGGGTTGGGTA	67
			GCTCTCAACTCATTCGTTGGAAGGC	TGAATGGGTTTGGGTTGGGTA	68
	Solyc03g006260	LOC101254062	TGTTACCAAGGTGCAAAGCAG	CCAAGAGGCTTGTCCAAAGTG	67
			CAGTACTATACACTTCATTCTTCAATAA	TAATAGTCCCAACCAAACAATTAATTA	61
	Solyc07g062700	LOC101263533	CCCAAATCGTTCTTCACTCTCC	AAACGACAGATGTGCACTGC	66
			CGGCGAATAGTTCTAAACATGAGAA	CGGGCATAAACAATAGCCAA	65
	Solyc12g014110	LOC101257045	AACTCCGATGGCATCTCTGG	CGACTAAAGTGTGCCGTAGC	67
CCX	*Solyc01g098800*	LOC101261070	CAATATTCTGGAATGGCATGGC	GTTCCAGACCAGTGATGGATG	65
	Solyc02g069710	LOC101265300	GGTGTTCAATCTTGTGTGTTCC	AAAGGAGTCGAAGAGCACATAC	65
			GGGTTTTCGATGCGTTTTGCTG	CGCCCACGCACTTCCATTACAAC	69
	Solyc07g006370	LOC101250521	CCAAACAACACTTTCACAATTCCC	TTCAGAGTCGACGTGCTTCC	66
			CATCTCTCTCACTATTATTATTAAAAAAAATGTC	GAGCTTCCTTTCAGAGTCGA	62
	Solyc07g042000	LOC101250709	CCCTGTTTAGTTTGTGGAGGG	CCACTTTGTGTTGCAACAGTAC	65
			CGCTTCCATGGCTCCCTATTTTC	CCCTGCTTTCACCCAAATGATGA	69
	Solyc09g072690	LOC101248713	CCCATTCCGTTTCAAGCCAA	CAACAAGAGCCCATAGTAAGCC	67
			CCTTATACATTTAGCTCTCTCTTCTCTTC	CATGTATACACTACATATAAACATCCTT GTC	64
MHX	*Solyc06g009130*	—	AAGATGACCTCGCTTGGAAAC	GATATCGCTCCATTTAAGCCCA	65

Table S2. List of primers used for expression analysis of *SlCaCA*.

Family name	Locus by ITAG 2.4	Gene symbol by NCBI	Primer sequence(5'→3') Foward	Reverse	Annealing temp(℃)
CAX	*Solyc03g123790*	LOC101267212	GGAATTGCATTTTGGCAAGT	CACCCTCGATGGCATCTACT	56.1
	Solyc06g006110	LOC101265339	TGCCAATAGTGGGAAATGCAGCAG	TTGAGAAGCTGACCCCAATGCAAC	58.2
	Solyc07g056110	LOC101252790	GTTGGAAATGCAGCTGAACA	TGGGCTTGTCCTGTATAGCC	56.1
	Solyc09g005260	LOC101254519	GTGGTTCCCTTGTGTGTGATTG	TCCTGTAGAGTGAAAGCTGTGAC	58.2
	Solyc12g011070	—	GATGGCCGAGGAAGAGGATCATA	CGGATGCTCCTTCTACGGTATTAAC	57
	Solyc12g055750	LOC101250779	TGAGTCAGCTCATATGGCGTCGC	TGTAGCAGTATCGCCAATGGTCCA	62.7
NCL	*Solyc02g077270*	LOC101251749	GCTCTCAACTCATTCGTTGGAAGGC	TGCATGCCGTTGTACATGATGCAG	60.8
	Solyc03g006260	LOC101254062	CAAGCTACGTATGGCCAATGTGACC	AGAGTAGCCCCAAAGATGCCTGTAC	58.2
	Solyc07g062700	LOC101263533	AGGAAACAAGGAGAGAGCACAG	GAAGTTCCATGTTAGACCTCGGG	57
	Solyc12g014110	LOC101257045	GGTCGCCGTTTCATCAGTGACGA	GTTCACAACTCTCCTCTTCACCTCCG	58.2
CCX	*Solyc01g098800*	LOC101261070	CGACCAGCAATTTCACTGACTCCAA	TCTGGCTTGACCTCCCCTAACTG	58.2
	Solyc02g069710	LOC101265300	CGGGTCGGATGCTGATGATTTGG	CGCCCACGCACTTCCATTACAAC	58.2
	Solyc07g006370	LOC101250521	GGTGCCTTTGTTGGTGGATGAGG	CCCCACAACAAAGCCCAATAACACC	62
	Solyc07g042000	LOC101250709	TTAGGGATCGCGAGACTAGTTTG	CTTGTAGCTGCAGCAATCCATC	58.2
	Solyc09g072690	LOC101248713	ACTCCTATAGACGCGGCCTCAAC	CCTCCAAGAAGCCAAATCCACAAACA	60.8
MHX	*Solyc06g009130*	—	CCGGTCCATGGAGAGTGTTGTCA	CCAACGTTTGTCCTGTGCATAGGC	58.2
Ubiquitin 3	*Solyc01g056940*	—	ACTCTTGCCGACTACAACATCCA	AACTGCAACACAGCGAGCTTAAC	57

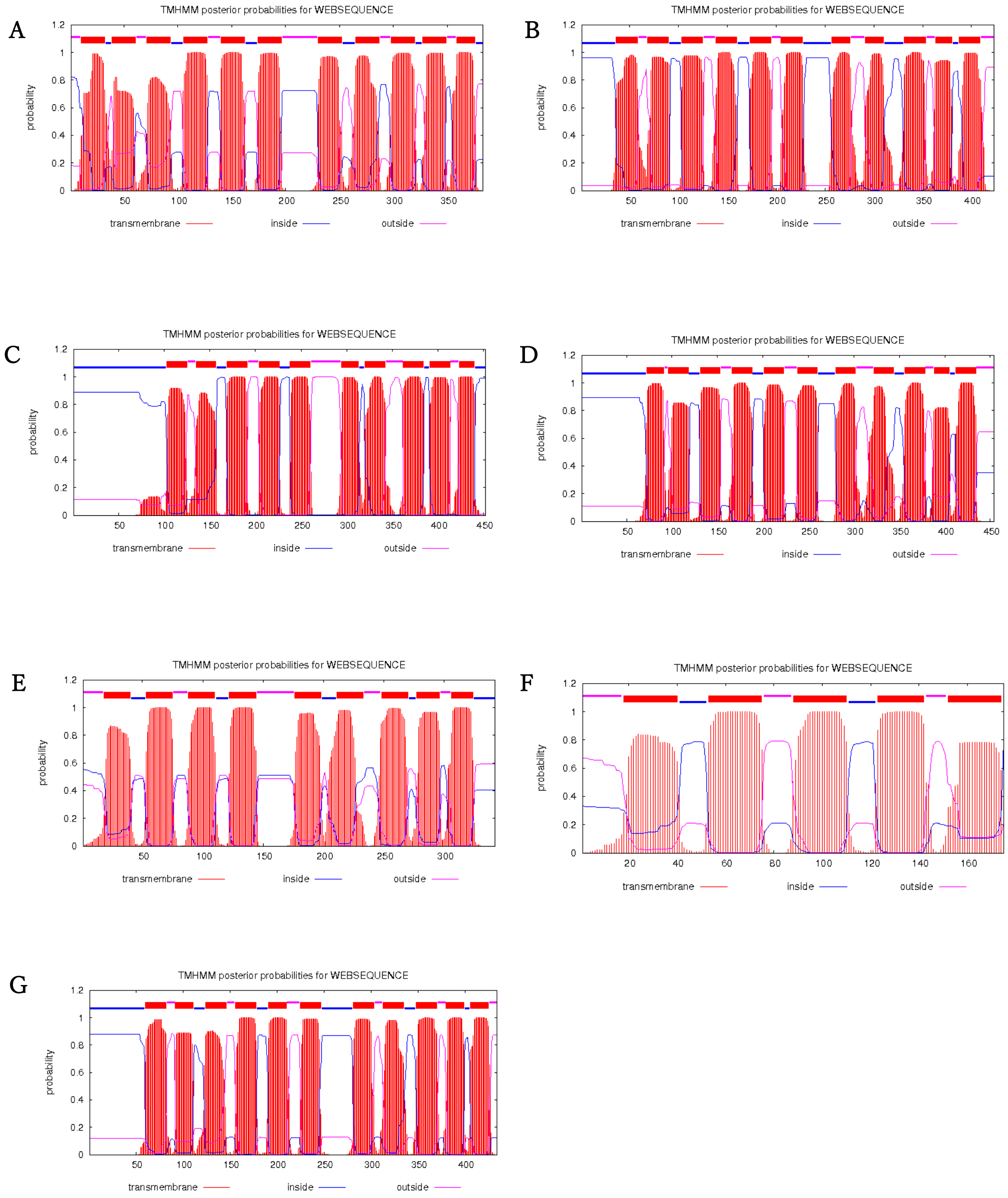

Figure S1. Transmembrane domains of SlCAX searched using TMHMM. **A**) Solyc03g123790, **B)** Solyc06g006110, **C)** Solyc07g056110, **D)** Solyc09g005260, **E)** Solyc12g011070a, **F)** Solyc12g011070b, c, d, **G)** Solyc12g055750.

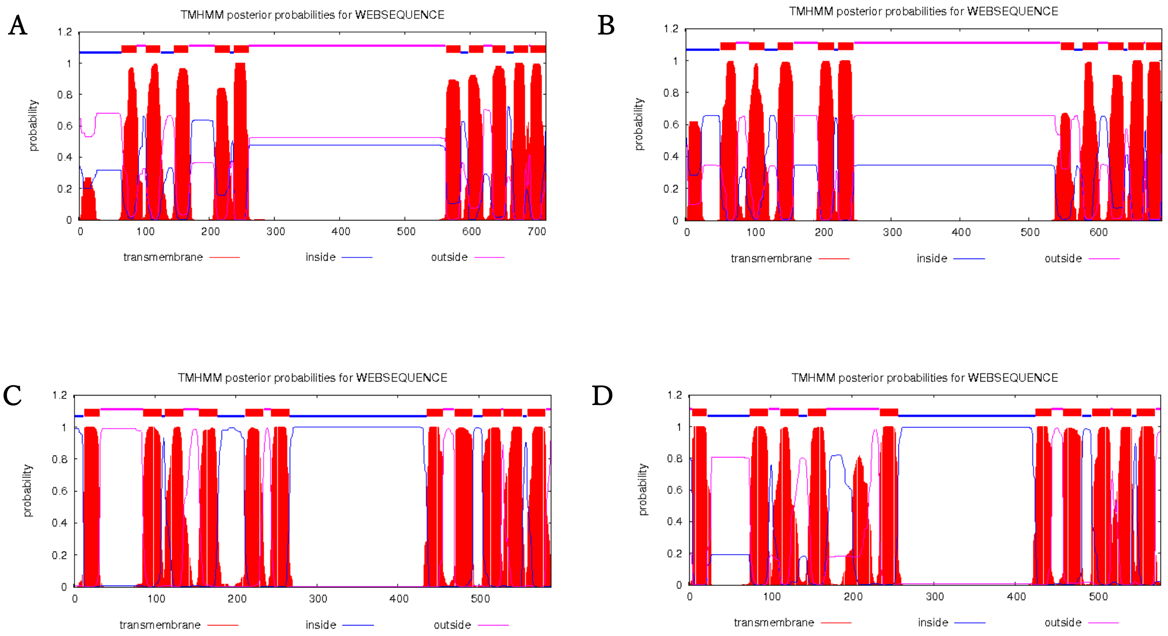

Figure S2. Transmembrane domains of SlNCL searched using TMHMM. **A)** Solyc02g077270, **B)** Solyc03g006260, **C)** Solyc07g062700, **D)** Solyc12g014110.

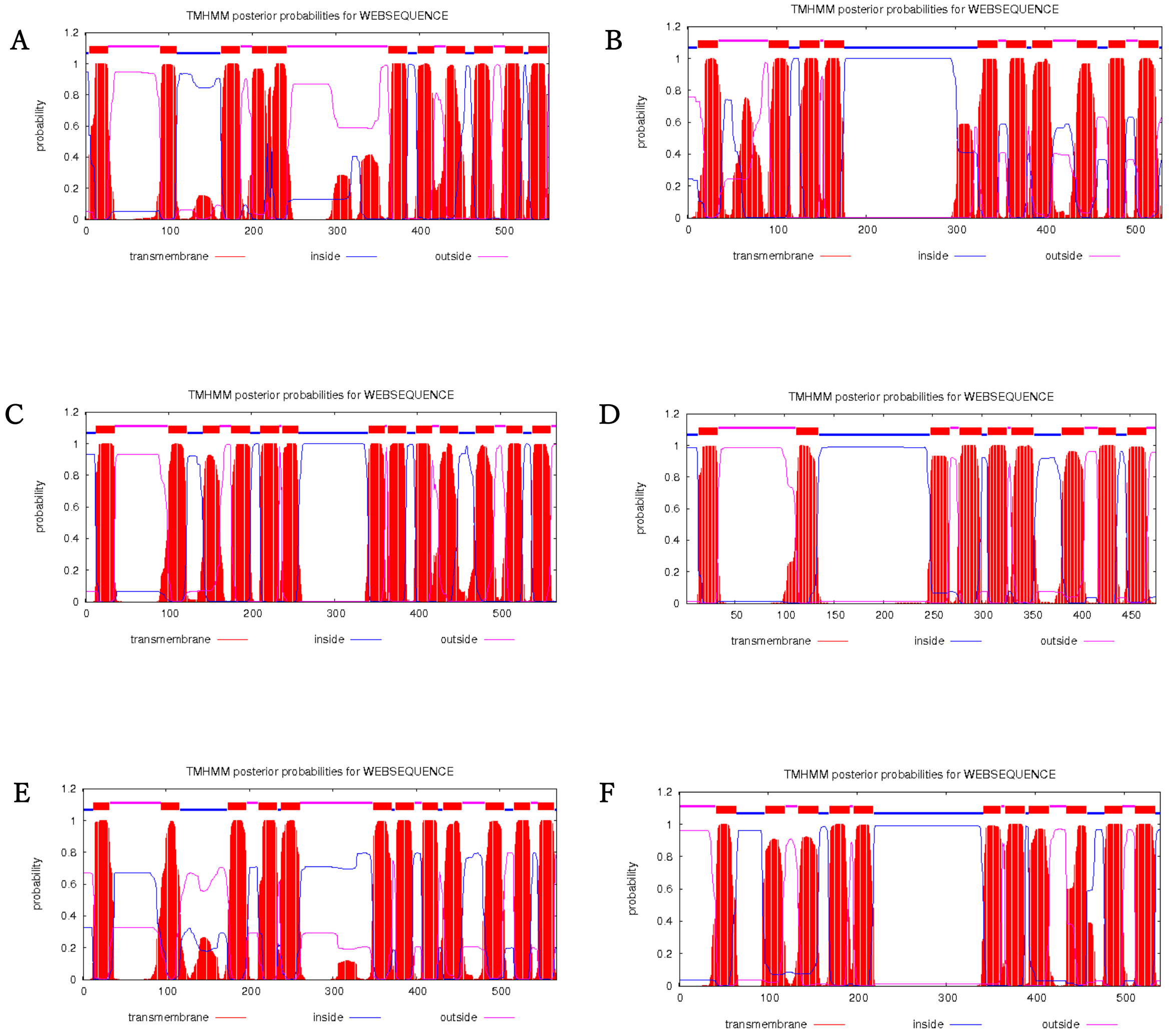

Figure S3. Transmembrane domains of SlCCX and SlMHX searched using TMHMM. **A)** Solyc01g098800, **B)** Solyc02g069710, **C)** Solyc07g006370, **D)** Solyc07g042000, **E)** Solyc09g072690, **F)** Solyc06g009130.

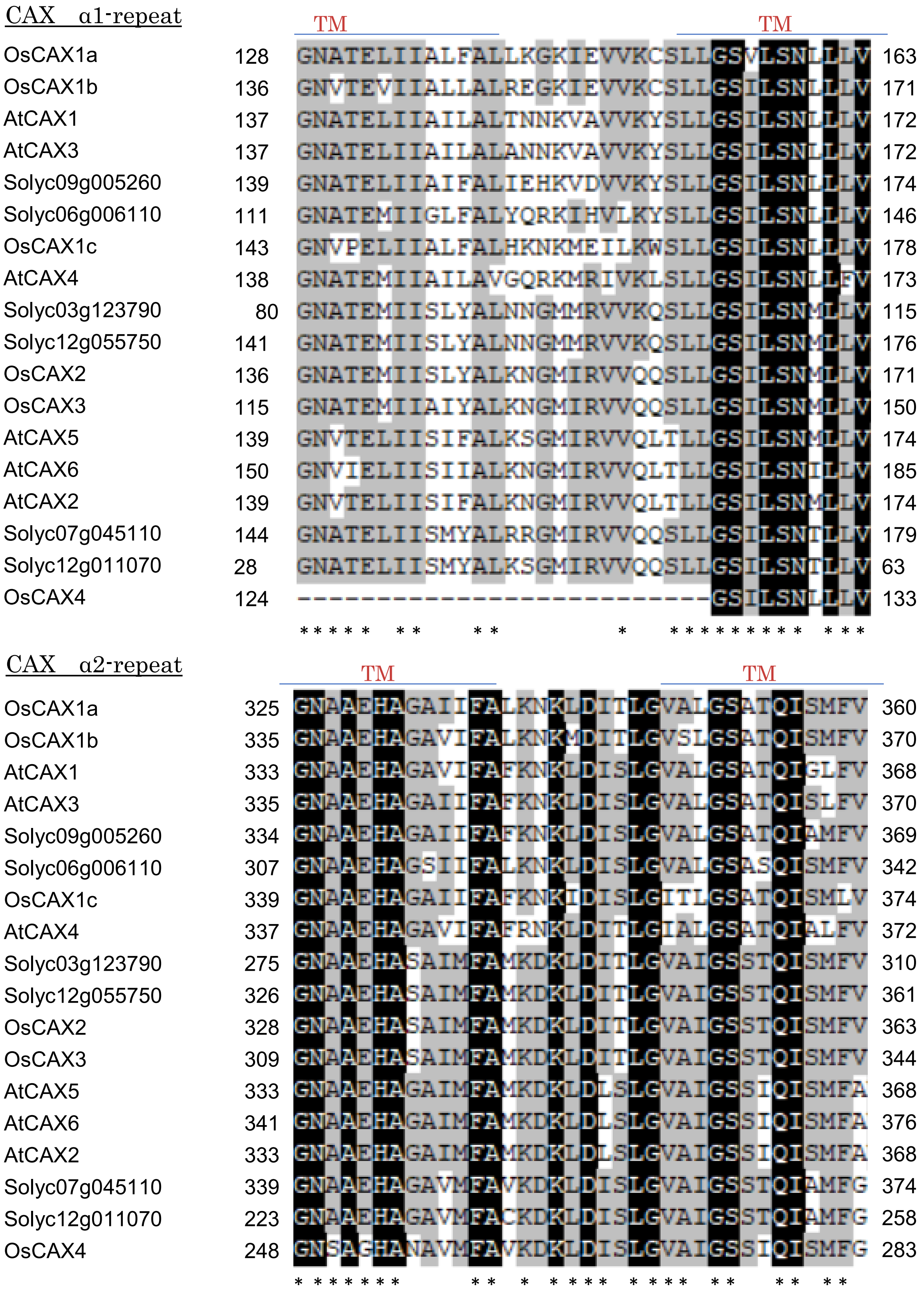

Figure S4. Multiple alignments of conserved α1-repeat and α2-repeat regions in CAX proteins. Amino acid sequence alignment was performed by ClustalW2 using CAX protein sequences from tomato, *Arabidopsis*, and rice. Asterisks and "TM" indicate identical amino acids among tomato CAXs and transmembrane domains, respectively.

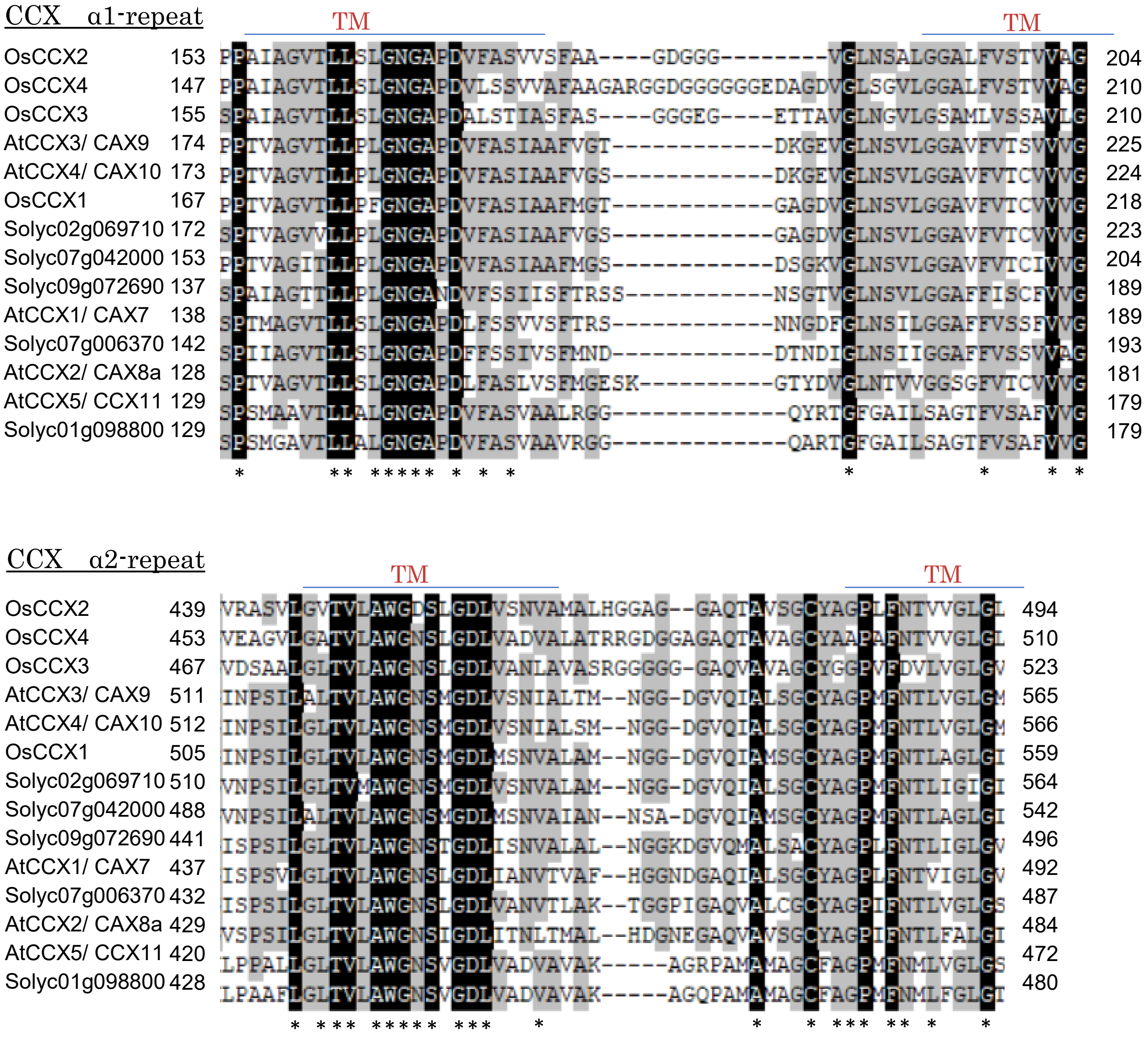

Figure S5. Multiple alignments of conserved α1-repeat and α2-repeat regions in CCX proteins. Amino acid sequence alignment was performed by ClustalW2 using CCX protein sequences from tomato, *Arabidopsis*, and rice. Asterisks and "TM" indicate identical amino acids among tomato CAXs and transmembrane domains, respectively.

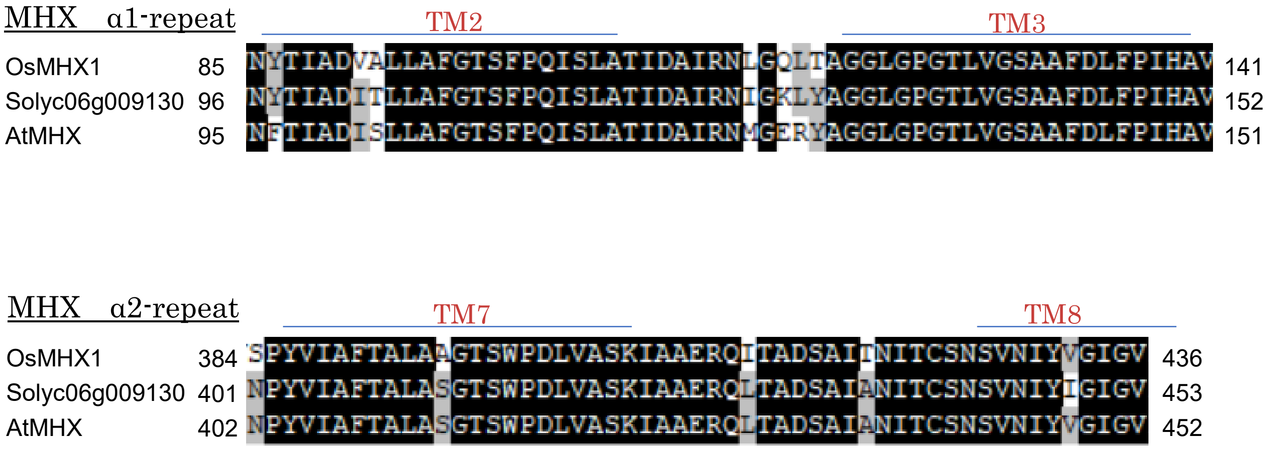

Figure S6. Multiple alignments of conserved α1-repeat and α2-repeat regions in MHX proteins. Amino acid sequence alignment was performed by ClustalW2 using MHX protein sequences from tomato, *Arabidopsis*, and rice. TM, transmembrane domains.

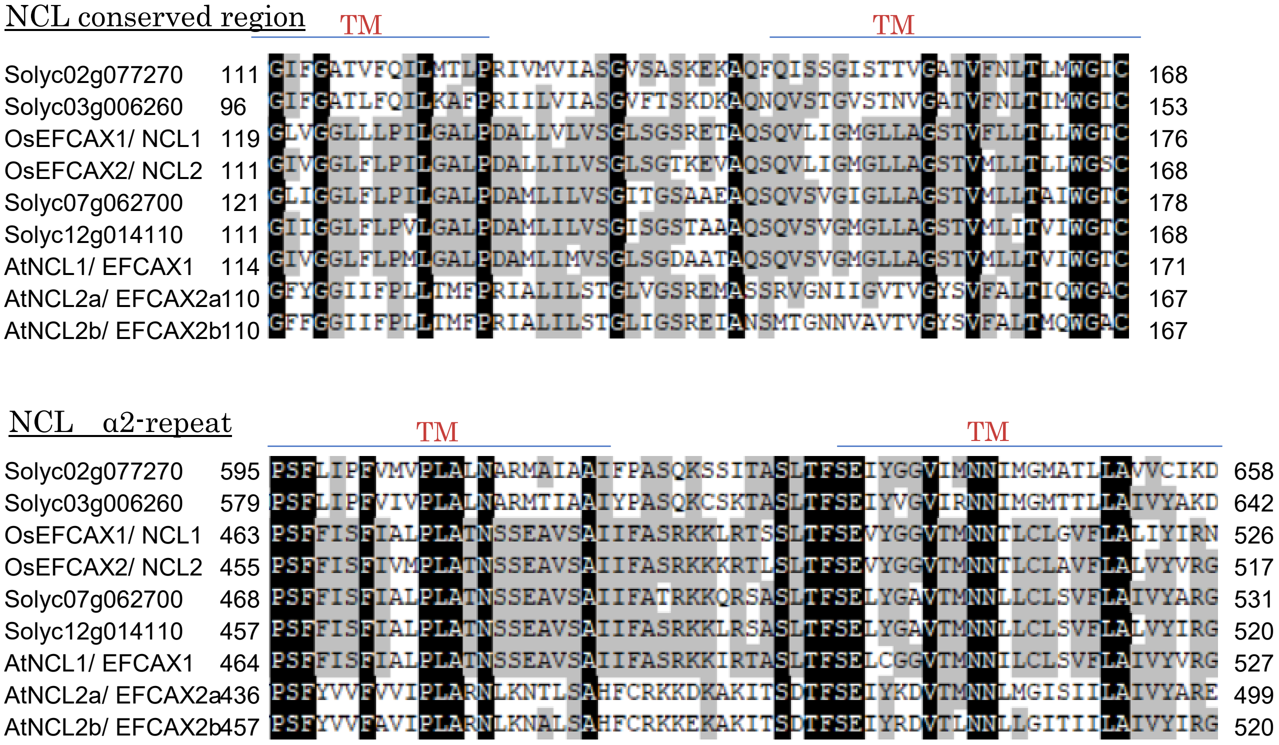

Figure S7. Multiple alignments of the conserved region, which may correspond to α1-repeat, and α2-repeat region in NCL proteins. Amino acid sequence alignment was performed by ClustalW2 using NCL protein sequences from tomato, *Arabidopsis*, and rice. Asterisks and "TM" indicate identical amino acids among tomato CAXs and transmembrane domains, respectively.
